# Development of Antiepileptic Drugs throughout History: From Serendipity to Artificial Intelligence

**DOI:** 10.3390/biomedicines11061632

**Published:** 2023-06-03

**Authors:** María Gabriela Corrales-Hernández, Sebastián Kurt Villarroel-Hagemann, Isabella Esther Mendoza-Rodelo, Leonardo Palacios-Sánchez, Mariana Gaviria-Carrillo, Natalia Buitrago-Ricaurte, Santiago Espinosa-Lugo, Carlos-Alberto Calderon-Ospina, Jesús Hernán Rodríguez-Quintana

**Affiliations:** 1 Pharmacology Unit, Department of Biomedical Sciences, School of Medicine and Health Sciences, Universidad del Rosario, Bogotá 111221, Colombia; maria.corrales@urosario.edu.co (M.G.C.-H.); sebastian.villarroel@urosario.edu.co (S.K.V.-H.); santiago.espinosal@urosario.edu.co (S.E.-L.); carlos.calderon@urosario.edu.co (C.-A.C.-O.); 2 Semillero de Investigación en Neurociencia, Semineuros, Universidad del Rosario, Bogotá 111221, Colombia; 3 Neuroscience Research Group (NeURos), NeuroVitae Center for Neuroscience, School of Medicine and Health Sciences, Universidad del Rosario, Bogotá 111221, Colombia; leonardo.palacios@urosario.edu.co (L.P.-S.); mariana.gaviria@urosario.edu.co (M.G.-C.); 4 School of Medicine and Health Sciences, Universidad del Rosario, Bogotá 111221, Colombia; natalia.buitragor@urosario.edu.co; 5 Research Group in Applied Biomedical Sciences (UR Biomed), School of Medicine and Health Sciences, Universidad del Rosario, Bogotá 111221, Colombia; 6 Fundacion CardioInfantil—Instituto de Cardiologia, Calle 163a # 13B-60, Bogotá 111156, Colombia; 7 Hospital Universitario Mayor Mederi, Calle 24 # 29–45, Bogotá 111411, Colombia

**Keywords:** epilepsy, history, antiepileptic drugs, artificial intelligence, drug development

## Abstract

This article provides a comprehensive narrative review of the history of antiepileptic drugs (AEDs) and their development over time. Firstly, it explores the significant role of serendipity in the discovery of essential AEDs that continue to be used today, such as phenobarbital and valproic acid. Subsequently, it delves into the historical progression of crucial preclinical models employed in the development of novel AEDs, including the maximal electroshock stimulation test, pentylenetetrazol-induced test, kindling models, and other animal models. Moving forward, a concise overview of the clinical advancement of major AEDs is provided, highlighting the initial milestones and the subsequent refinement of this process in recent decades, in line with the emergence of evidence-based medicine and the implementation of increasingly rigorous controlled clinical trials. Lastly, the article explores the contributions of artificial intelligence, while also offering recommendations and discussing future perspectives for the development of new AEDs.

## 1. Introduction

Epilepsy is a complex neurological disorder that can be acquired as a result of brain injury from trauma, stroke, infections, tumors, or even from genetic mutations in proteins controlling brain excitability, ion channels, or neurotransmitter genes. It affects ~50 million people worldwide and has a lifetime prevalence of ~1% [1,2,3]. Within its range of presentations, seizures can be considered the ultimate clinical manifestation of epilepsy. As such, discovering drugs that can control seizure spread is of utmost importance.

### 1.1. History of Epilepsy

Although under different names and etiologies, epilepsy and seizures can be traced back as far as the Mesopotamian civilization more than 3000 years ago [4]. One of its ancient books, the *Sakikku*, which translates to “all diseases”, gathered diagnostic information for different diseases, among which epilepsy and seizures can be recognized with a description of an unconscious man whose neck is turned, whose extremities are tense, and whose eyes are wide open; they named it *miqtu*, meaning “the falling disease” [5]. Throughout the centuries, different civilizations have each depicted and described what nowadays can be defined as epilepsy. Many civilizations believed epilepsy had a magical or occult origin: these included the Akkadian culture, and authors of the *Sakikku*, who directly related it to Sin, the god of the moon. This magical conception persisted throughout different civilizations until around the 5th century BC when the Hippocratic school of medicine challenged the established belief [6]. Said school stated that epilepsy, which derives from the Greek word *epilambanein*, meaning “to take hold”, was a result of an overflow of phlegm in the brain and argued that the disease, which was called at the time “the sacred disease”, was no more divine that other diseases, meaning it had a natural origin and, thus, a possible cure [7].

### 1.2. Definitions

In 2005, the International League Against Epilepsy (ILAE) conceptually defined epilepsy as an “enduring predisposition of the brain to generate epileptic seizures, with neurobiological, cognitive, psychological, and social consequences. The definition requires the occurrence of at least one epileptic seizure” [8]. However, this definition was not easily applied in the clinical field, which is why the ILAE presented the practical or operational definition of epilepsy stating that a patient is diagnosed with epilepsy if they meet any one of the following criteria: (1) at least two unprovoked (or reflex) seizures occurring >24 h apart; (2) one unprovoked (or reflex) seizure and a probability of further seizures similar to the general recurrence risk (at least 60%) after two unprovoked seizures, occurring over the next 10 years; (3) and diagnosis of an epilepsy syndrome [9]. Additionally, the ILAE’s Task Force Report states that epilepsy should be considered as resolved when the patient has not presented seizures for the past 10 years with no seizure medication for the past 5 years; or when the patient presents with an age-dependent epilepsy syndrome and the patient has passed the applicable age [10].

### 1.3. Epidemiology

Epilepsy is one of the most common chronic neurological diseases worldwide and affects people of all ages, races, and socioeconomic conditions. In 2016 there were 45.9 million people with active epilepsy globally, either of idiopathic or secondary nature. A systematic review and meta-analysis of international incidence performed by Fiest et al. [11] reported a pooled incidence of 61.4 per 100,000 people, with a higher incidence in low- and middle-income countries (LMIC) than in high-income countries (HIC) (139 per 100,000 vs. 48.9 per 100,000, respectively). It is believed that this difference in incidence originates in structural and socioeconomic gaps, as well as higher exposure to risk factors for known etiologies of epilepsy such as infections, traumatic brain injuries, and deficient perinatal care, among others [12]. When it comes to age, a bimodal distribution has been described: incidence is higher during the first two decades and after the eighth decade of life. Said bimodal distribution might be explained by higher numbers of epileptic syndromes occurring during infancy, and epilepsy as a result of stroke, tumors, and dementia in the elderly population [9].

As mentioned in the definition, epilepsy has an intrinsic social and psychological component, not only for the patient but also for their family or caregiver. A systematic analysis of the Global Burden of Disease Study 2016 concluded that although there has been a substantial decrease in the mortality and the DALY rates between 1990 and 2016, there is still a significant treatment gap which might explain the notable difference in fatality and disease severity when comparing LMICs and HICs [12]. According to the World Health Organization (WHO), despite the availability of effective and low-cost antiepileptic medication, more than 75% of people in LMICs do not have access to treatment. They also affirm that 70% of people with epilepsy worldwide could resolve their disease with adequate use of cost-effective antiepileptic medication [13]. A study performed by Orozco et al. [14] reported a prevalence of 11.3 cases per 1000 people in Colombia, 37% of whom had a drug-resistant form of epilepsy. The use of phenobarbital as an AED has been discouraged in developed countries mostly because of its neurotoxicity, which manifests as sedation, mood changes, and behavioral and cognitive alterations, among other symptomatology [15,16]; however, in the previously mentioned study, 28% of patients were receiving phenytoin and 14% phenobarbital as monotherapy. Ethical discussions have arisen from the implications phenobarbital use might have, bearing in mind that one of the strongest reasons for using it is its cost-effectiveness and low cost compared to other AEDs. As Nimaga et al. pointed out, “sometimes the choice is not between phenobarbital and a new medication but between phenobarbital and no treatment at all” [17]. Nevertheless, ethical debates and considerations go beyond the scope of this article.

### 1.4. Epileptogenesis

A plethora of etiologies have been identified as the causal agents of epilepsy; however, there is one thing that remains common to all of them; an initial brain insult is always described as the culprit. The most commonly described causes are structural (such as epilepsies resulting from stroke or traumatic brain injury), infectious diseases, and immunologic and genetic conditions [18]. Epilepsies that are not correctly classified into one of the previously mentioned categories are said to be of idiopathic origin and are still uncharted territory. During epileptic seizures, hyperexcitability and synchronous-firing neurons result in a cascade of molecular events which act as perpetuators of neuronal damage. Throughout the years, these repercussions have been broadly studied and a common actor has risen; oxidative stress (OS) has remained one of the main highlights in epileptogenesis and chronic neurodegeneration.

Brain tissue’s metabolic demand is one of the greatest in the human body, accounting for approximately 20% of oxygen metabolism [19]. As it is known, cellular metabolism results in reactive oxygen (ROS) and nitrogen species (RNS) such as superoxide (O^2−^), hydrogen peroxide (H_2_O_2_), and hydroxyl radical (OH), among others. Under physiological conditions, ROS can play a crucial role in cell signaling in nervous, skeletal, and cardiovascular systems among others; however, overproduction has a deleterious effect [20]. Antioxidant enzymes such as catalase, superoxide dismutase, and glutathione peroxidase along with non-enzymatic mechanisms such as vitamins C and E are some of the buffering mechanisms the organism has to counteract an increase in ROS, or what is otherwise known as oxidative stress. The brain’s high metabolic demand and low antioxidant enzyme activity render it particularly susceptible to OS detrimental consequences [21,22].

The relation between OS and epilepsy has been widely studied and the questions mainly asked remain: whether neurological damage resulting from OS acts as a causative agent or rather acts as a perpetuator of it. Seizure-induced DNA oxidative damage demonstrated by 8-hydroxy-2′-deoxyguanosine (8-oxo-dG), a guanine adduct resulting from oxidation of mitochondrial DNA (mtDNA) bases has been proven to be increased in kainite-induced seizure animal models [23]. As 8-oxo-dG acts as an indirect measure of oxidative mitochondrial DNA (mtDNA) damage, explained by the proximity that exists between the mitochondrial electron transport chain (the main source of ROS) and mtDNA, these findings are consistent with the premise that seizure-induced ROS are perpetuators of neurological damage [24]. Additionally, a direct correlation between mitochondrial antioxidant agents and neurodegeneration strengthens the ROS involvement hypothesis, wherein said correlation was demonstrated by animal studies in which transgenic mice with overexpression of superoxide dismutase (SOD) were unsusceptible to seizure-induced neurodegeneration, whilst partially deficient SOD mice had a higher susceptibility to seizures and neurodegeneration arising from them [18].

Moreover, a direct response to antioxidant therapy in both animal models and clinical trials further supports the previously mentioned hypothesis; clinical evidence from a study performed in 1984 by Kovalenko demonstrated substantial benefits to a cohort of drug-resistant epileptic patients to whom alpha α-Tocopherol (Vitamin E isoform with the highest in vivo biological activity) was administered [25]. Positive outcomes such as reduction of seizure frequency and improvement in pathological electroencephalogram findings (EEG) were documented; said study has been reproduced by different authors maintaining the initial findings and corroborating its premise [26].

Glutamate build-up and the consequent polarization–depolarization cycle it produces has been one of the cornerstones described in the pathophysiology of epilepsy; as previously stated, epileptic seizures arise from a disbalance between excitatory and inhibitory mechanisms. Glutamate, the most commonly found excitatory neurotransmitter in the brain, plays a crucial role in epileptogenesis and its accumulation leads to what is known as excitotoxicity. Aberrant connectivity and sporadic depolarization occurring during seizures lead to high glutamate concentrations in the postsynaptic cleft and the extracellular space. Under normal circumstances, astrocytes are able to reuptake excess glutamate via excitatory amino acid transporter-2 (EAAT-2) and convert it to glutamine with the help of glutamine synthetase (GS); however, when intracellular calcium concentration within astrocytes rises, the mechanism is overridden, thus further increasing glutamate extracellular concentration [27,28,29]. Furthermore, a study performed by Eid et al. [30] found a deficiency in GS concentrations in surgically resected hippocampi from mesial temporal lobe epilepsy (MTLE) patients, thus confirming excitotoxicity’s major role in epilepsy’s pathophysiology [31].

Finally, as extracellular glutamate concentrations keep rising and N-methyl-D-aspartate (NMDA) receptors (glutamate’s metabotropic receptor) are activated, intracellular calcium concentrations begin rising as well. Elevated intracellular calcium concentrations are detrimental to cellular functionality and will eventually result in cellular death [27]. Some of the mechanisms through which this outcome is reached are the overproduction of ROS and RNS, nitric oxide synthase activation, protease activation, and generation of mitochondrial pores through which ROS, RNS, and contents such as cytochrome C will be leaked, activating caspases and nucleases leading to a cascade of events, resulting in cellular death. Elevated calcium influx will also lead to overstimulation of nitric oxide (NO) synthetase activity and the formation of more NO molecules which, combined with superoxide molecules, result in peroxynitrite ions causing lipid peroxidation [32].

## 2. History of Antiepileptic Drugs and Serendipity

Epilepsy is one of the most ancient medical conditions to be documented, as well as one of the most stigmatized diseases [33,34]. There is evidence of trepanned skulls in different parts of the planet, suggesting that prehistoric physicians performed this type of surgery on people who probably suffered from epilepsy, some forms of headaches, and/or mental disorders [33,34,35]. Since the first documentation of epilepsy circa 3000 BC, more than four thousand years went by before the appearance of what seemed to be the first effective antiepileptic drug—potassium bromide. Charles Locock (1799–1875), obstetrician and physician to Queen Victoria, demonstrated in 1857 the beneficial effect of this drug in patients with catamenial seizures. Its efficacy was rapidly noted as it demonstrated improvements in a significant number of patients with epilepsy, regardless of etiology or gender [36].

### Serendipity: Phenobarbital and Valproic Acid

In 1912, half a century after the discovery of potassium bromide’s antiepileptic properties, a second drug was discovered by Alfred Hauptmann (1881–1948), a German psychiatrist and neurologist. Phenobarbital was first used as a sleep inducer; however, its beneficial antiepileptic effect was also documented [37]. A third molecule was serendipitously discovered 50 years later in the year 1962. Valproic acid was used as a solvent in various pharmacological products, among which were various cough syrups. Epileptic patients presenting with a cough who were given these cough syrups showed a significant decrease in the number of seizures, and thus valproic acid’s antiepileptic properties became an object of study. In France, the Meunier brothers decided to test it as an antiepileptic, demonstrating its great anticonvulsant efficacy. It was first marketed in the year 1967, causing its use to skyrocket [38].

Although these drugs represented a very important improvement for epileptic patients, some of their side effects such as sedation and high addictiveness caused difficulties among patients, thus further prompting the search for new molecules [39].

## 3. Preclinical Models

**Figure 1:**
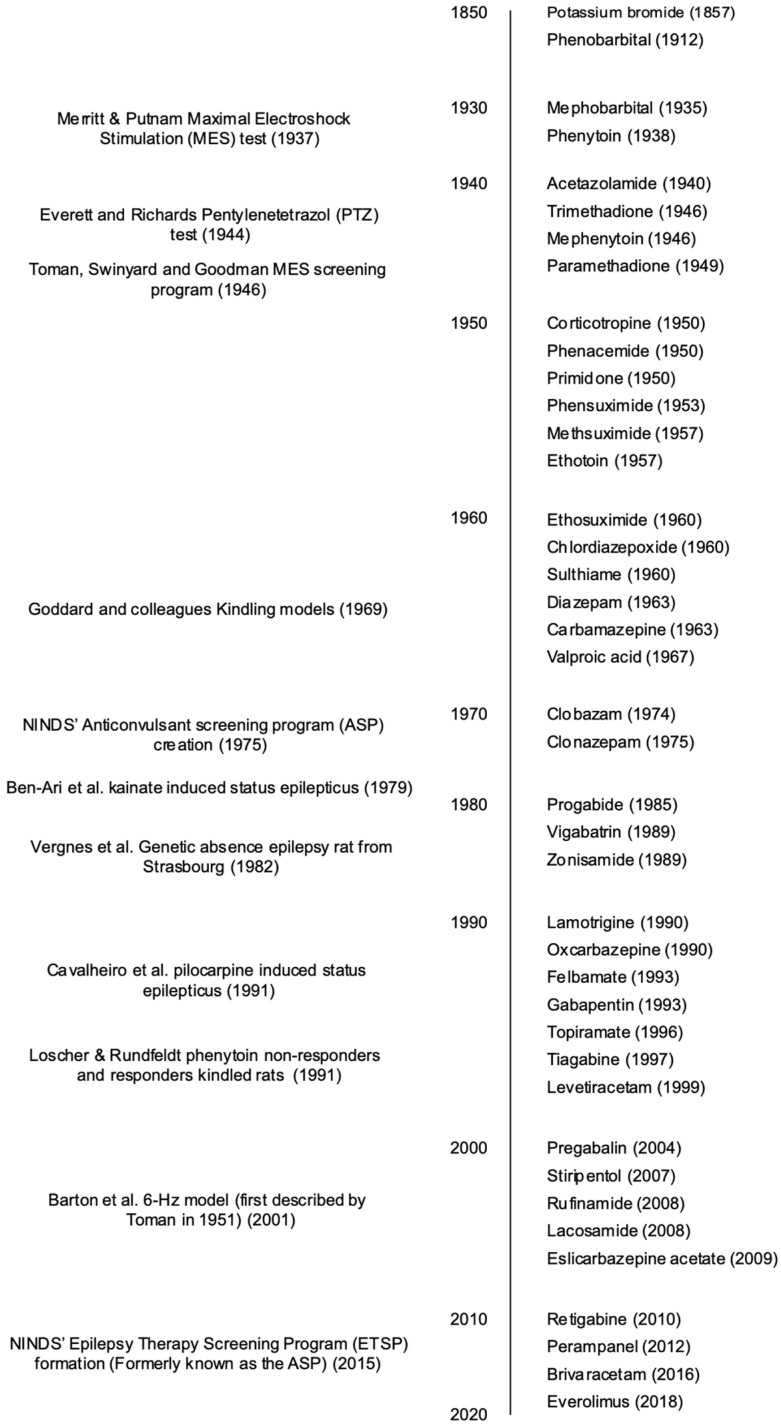
Milestones in the development of preclinical models for discovering and developing antiepileptic drugs (AED), as well as the introduction of different AEDs to the American, European, or Japanese markets. Various other models and AEDs are not shown in the figure. NINDS: National Institutes of Neurological Disorders and Stroke. Adapted from [57].

Ever since the introduction of maximal electroshock stimulation in 1937, the identification of new antiepileptic drugs (AED) for the treatment of epilepsy and seizures has been done in vivo on animal seizure models [40]. Throughout the years, a variety of new rodent models have been added to the arsenal of research protocols, each one accounting for different types of epileptic and seizure models, in order to better identify potential new AEDs and their distinct mechanisms of action, tolerability, and therapeutic index, as well as different programs focused on discovering and developing AEDs such as the Anticonvulsant Screening Program (ASP), known today as the Epilepsy Therapy Screening Program (ETSP). However, because of human epilepsy and seizure heterogeneity, there is still a need to find new etiology-oriented models that account for specific types of seizures, and to introduce new AEDs for the ~30% of patients who have failed to appropriately control their epilepsy and seizures with currently available treatments [1,41,42]. In this section, we aim to present a brief overview of the three most used and validated rodent models, as well as determine their different approaches and applicability regarding human epilepsies and seizures, together with their advantages and disadvantages. A comprehensive timeline of the most important milestones in preclinical AED models is provided in Figure 1.

### 3.1. Maximal Electroshock Stimulation (MES) Test

This model was first introduced by Merritt and Putnam in 1937. It consists of electrically evoking acute seizures, and it is used to characterize the response of cats treated with phenytoin [43]. At the present time, MES is performed on mice and rats; however, other animal species are also susceptible to electrically induced acute seizures and can be used to study potential new AEDs [44]. Regardless of the animal species and current intensity, MES seizures are induced by administering a shock through corneal or ear clip electrodes on a previously healthy and neurologically intact subject [40,44]. When using corneal electrode implants, a saline solution of 0.9% with local anesthetic must be applied to the eyes to improve electrical conductance and implant tolerability. For their part, ear clips must be placed with electrode gels to improve conductance. Additionally, to guarantee replicability among test subjects, electrodes are placed in the same place and at equal pressure. These electrodes are then connected to a device capable of administering sufficient electrical current to evoke a seizure [44]. Pharmacology testing is performed by administering the potential AED, delivering the same current intensity to all test subjects, and recording the extent of protection, among other outcomes [40,45,46]. Likewise, a threshold test can be done by administering progressively increasing currents in a stepwise manner if no endpoint (or seizure) is observed, starting from a known established threshold [47].

MES-induced seizures are electrophysiologically consistent with human seizures, rendering this test highly reproducible. This model, therefore, represents primary generalized tonic–clonic seizures and can be used for the initial identification of potential AEDs that can stop seizure spread or increase seizure threshold [3,40,44]. As an important note, however, some clinically effective AEDs such as levetiracetam, vigabatrin, and others do not show efficacy in the MES test; hence, testing for new potential AEDs by the MES test alone is not sufficient to identify antiseizure efficacy and other tests should therefore be used [1,40,44]. Likewise, because it does not generate focal seizures, the MES test cannot evaluate agents that target and affect seizure focus directly [1,40].

### 3.2. Pentylenetetrazol (PTZ) Induced Test

Although its mechanism of action is not fully understood, it is generally accepted that PTZ is a non-competitive antagonist of the gamma-aminobutyric acid (GABA)(A) receptor complex; hence, the PTZ molecule has epileptogenic properties [48]. According to this principle, it can be used to test potential AEDs. Regardless of the test subject—usually mice or rats—a certain dose of PTZ is administered, either intravenously (ivPTZ) or subcutaneously (scPTZ), a threshold is established once seizure activity is observed, and seizure patterns are recorded. It is important to note that PTZ-induced seizures also evoke generalized clonic and tonic seizures; however, these differ from MES-induced seizures in the observed pattern. MES seizures start with a tonic phase followed by a clonic phase, whereas PTZ results in a clonic phase first, followed by usually infrequent tonic phases [30]. This model was first validated by Everett and Richards in 1944 [49].

Pharmacology testing using PTZ follows the same principles as the MES test: the potential AED is administered, PTZ is injected at threshold doses, and outcomes are recorded. The ivPTZ test is preferred to determine whether the compound affects the seizure threshold, by using an infusion protocol starting at a known and established threshold dose and recording outcomes during infusion [44]. On the other hand, the scPTZ test can be useful to research AED response and outcomes on generalized non-convulsive myoclonic and generalized spike–wave seizures [42]. However, like the MES test, there are clinically effective AEDs that are not effective when tested on PTZ-induced seizures, so this test is not sufficient when used alone to determine antiseizure efficacy.

### 3.3. Kindling Models

First described by Goddard and colleagues in 1969 [50], kindling is defined as a process in which daily repetitive administration of subconvulsive stimuli leads to brain alteration and development of focal seizures, followed by secondarily generalized seizures as a result of the recruitment of different brain areas [2,51]. Hence, this model mimics epileptogenesis and can be used to test the efficacy of AEDs against focal and secondary generalized seizures, as well as their long-term efficiency to control epileptogenesis [44].

Kindling models vary according to the stimuli (chemical, electrical, implant, corneal) [52,53], the kindled brain area, and the expected outcome (anti-epileptogenesis or anticonvulsant activity). The most used and well-known model is kindling of the mesial temporal lobe or the basolateral amygdala in rats with an electrical stimulus [2], during which the exact sequence of behavioral manifestations (both epileptic/seizure stages and behavioral comorbidities such as anxiety or cognitive deficits) are recorded [42], as these can drastically change depending on the brain area affected. Ergo, this model requires precise preparation and time, especially when testing for chronic effects [44,52]. Unlike the other tests, this model proved the efficacy of different AEDs, including tiagabine, vigabatrin, and levetiracetam, as well as demonstrating the lack of clinical efficiency of NMDA antagonists, meaning this model can complement the other tests [1,40,44]. It is cautioned, however, that the antiepileptogenic or disease-modifying activity identified by this model has not been yet validated by human clinical trials [44].

### 3.4. Specific Models

Although the models explained above have been validated as useful assays to test for potential AEDs, there has been a need to implement new models to account for specific types of epilepsy and seizures, such as in the pediatric population, those caused by genetic mutations, drug-resistant epilepsy, status epilepticus, among others. However, these go beyond the scope of this article.

### 3.5. Contrasting Different Models

Both MES and PTZ models evoke acute generalized seizures with different patterns and are often used as a first-line test to evaluate the antiseizure efficacy of potential AEDs, specifically their protection against seizure spread or threshold increase on generalized seizures. These tests are easily reproduced on healthy neurologically intact subjects, require little preparation compared to kindling models, and multiple studies can be conducted in short periods of time, as only acute responses are evaluated, providing useful insight and data for further research on more specific models. However, negative results in the MES or ivPTZ/scPTZ test do not automatically rule out mechanistically novel molecules that target different pathways, and other tests should be employed to further study different mechanisms of action. For example, tiagabine and vigabatrin are not effective on the MES test but are on the PTZ tests, while levetiracetam is not effective in either test, but it works against kindled focal seizures [1,40,44].

For their part, kindled models have proven valuable in discovering different AEDs with clinical efficacy and have provided results regarding antiepileptogenic and antiseizure efficacy in focal and spike–wave seizures, unlike the aforementioned tests, although drawbacks can be seen as these models require precise experimental logistics. As these mice or rats are implanted with stimulating/recording electrodes in different brain areas, and seizure patterns and behavioral stages are recorded precisely over a long period of time, several considerations must be taken into account when conducting these tests, some of them being: the adequate facilities and surgical resources required to implant these electrodes, the housing needed for multiple test subjects, and the time required to conduct studies for chronic effects of potential AEDs [44], in contrast to the acute models, which need significantly less time to evaluate response and outcomes. Hence, kindled studies are often restricted to late preclinical testing after promising AEDs have been found to have robust antiseizure activity, as they can be useful to complement results from other tests. A comparison of these three classic models is presented in Table 1.

By 1952 Swinyard and colleagues in the United States had identified a series of new hydantoins, diones, succinimides, barbiturates, and deoxy barbiturates when exhaustively exploring the cyclic ureide structure shared by both phenytoin and trimethadione, primarily using the MES and PTZ tests [54,55]. However, once ethosuximide was successfully marketed in 1960, almost all research was stalled, as the concept of approaching new molecules with different structures was seen as an expensive task with a very high failure rate [55], for which no unified workflow to facilitate testing and data management existed at the time, and there was little to no funding for new AED discovery and development. This sudden stop in research brought about a “drought” in new AED discovery for almost a decade in the United States, something which was noticed by Richard Masland, Director of the National Institute of Neurological Disorders and Stroke (NINDS), who appointed J. Kiffin Penry in 1967 and a few years later Roger J. Porter to the newly formed Section on Epilepsy at the NINDS, giving them the objective to create a better environment for discovering and developing new AEDs [55].

During his period at the NINDS, Penry created the Epilepsy Advisory Committee, which helped lay the groundwork for the anticonvulsant screening program (ASP), a program seeking to perform blinded screenings of compounds in a variety of acute and chronic seizure models, as well as in animals with epilepsy [54,55,56]. A request for a proposal was issued to major pharmaceutical companies and university departments to supply the ASP with novel chemical compounds. Whenever a screened compound had shown promising activity, it would be commented to the provider, where it would most often receive an additional test to further evaluate for potential antiseizure activity. It is approximated that by the year 2018, more than 32,000 novel compounds had been screened. Some major contributions the ASP provided were identifying potential in felbamate, topiramate, and lacosamide, among others [54,55]. Once Penry retired, the project was run by Porter, who took over and managed the Epilepsy Branch from 1979 to 1984, followed by Steve White, who was involved in the program from 1986 and was formally instituted as the director of the Anticonvulsant Drug Development (ADD) Program at the University of Utah in 2001 [55]. Of note is that in 2015 the ASP changed its name to the new Epilepsy Therapy Screening Program (ETSP), emphasizing the need to identify differentiated compounds to address the unmet medical needs of epilepsy, such as pharmacoresistant epilepsies [57].

Despite the high predictive values of these previous models and their utility in continuing to identify new effective treatments for patients with epilepsy and seizures, there is still a need to implement new models that account for etiology-oriented epilepsies, among which are drug-resistant epilepsy, acute and chronic seizure phenotypes such as absent epilepsies, pediatric encephalopathies, and genetic mutations [3]. To this end, several recent rodent models have tried to replicate and mimic specific known acquired and genetic etiologies of seizures to investigate potential and existing AEDs, such as the 6 Hz psychomotor model in rats [53], lamotrigine-resistant models, phenytoin-resistant models, and post-status epilepticus models, among others [1]. However, these are usually labor intensive, as replicating and reproducing specific etiologies, namely, mutations or drug resistance, can be challenging [44,52]. Therefore, there remains a substantial unmet need for patients with either drug-resistant epilepsy or difficult-to-control epilepsies; for instance, 25–40% of patients with focal epilepsy fail to achieve therapeutic goals with currently available AEDs [1,41].

### 3.6. Drug Safety

It is important to note that the aforementioned tests do not evaluate drug safety when used in humans; rather, these only serve as screening tests for antiepileptic activity in novel chemical compounds. Hence, it is necessary to conduct proper pre-clinical trials to further study potential adverse effects in humans, such as conducting toxicology studies in two different animal species, usually a rodent and a nonrodent, as the use of two or more unrelated animal species may increase the probability of detecting potential adverse effects in humans [58], or testing for more specific body systems, especially that of the central nervous system given the potential properties of AEDs, such as the rotarod test to evaluate for effects on neuromuscular coordination or assess for higher cognitive functions to detect impairment in learning, memory, attention, and executive functions [59]

Failure to account for drug safety can lead to adverse effects in humans; for example, different studies have detected an increased risk of teratogenicity when using AEDs during pregnancy, especially that of valproic acid [60], or the vigabatrin-induced retinal toxicity when used chronically [61]. The contrary can happen as well, where an adverse effect is found in animal studies, but no human case has been reported, meaning animal studies have limitations in accurately representing potential unwanted side effects in humans, as in, for example, the fact that valproic acid produces testis, epididymis, prostate gland, and seminal vesicles atrophy in Wistar rats, but not in humans [62].

Despite limitations, these should not discourage nor constrain any attempts in further testing for potential adverse effects detection. However, the adverse effects of these drug categories and their appropriate testing are beyond the scope of this article.

## 4. Clinical Trials for AEDs

For a concise historical review of the clinical development of AEDs, a search was conducted in PubMed using the names of individual AEDs as the MeSH term, combined with the term ‘Clinical Trial’ [Publication Type], employing the Boolean operator ‘AND’ (e.g., ‘Phenytoin’[MeSH] AND ‘Clinical Trial’ [Publication Type]). The article selection process was performed independently by two authors (clinical neurologists) following their own criteria. Any discrepancies were resolved through consensus by a third author (clinical pharmacologist). Strict filters were applied to refine the search results, ensuring the inclusion of solely clinical studies conducted on human subjects and published in either English or Spanish.

Clinical trials play a crucial role in confirming the safety and effectiveness of novel drugs, and it is essential for this process to adhere to ethical and scientific quality standards [63]. Human clinical trials have been conducted since ancient times, with accounts dating back to 500 BC in the Old Testament. The first recorded clinical study in history was carried out by Ambroise Paré in 1537. However, the design of clinical studies has evolved significantly over time [64]. In this section, we will provide a brief overview of the development of clinical studies in the field of AEDs throughout history. The initial antiseizure medications were approved without stringent regulatory requirements and did not undergo the three phases of clinical trials. Instead, following experiments with phenytoin in animal models, uncontrolled observational studies involving individuals with epilepsy were initiated [65,66]. Although the initial findings from these clinical observations demonstrated a reduction of the number of seizures, the observation period was relatively short (average of 4.3 months), the sample size was relatively small (200 subjects), and neither a control group nor a placebo was utilized. The first observational clinical studies on phenytoin were published in 1938, with regulatory approval occurring a year later [65].

After the discovery of phenytoin and until the 1950s, there was an increase in clinical trials of novel AEDs (to 15% during the 1930s and up to 56% during the 1950s) [67]. In the period until 1970, 250 studies were published evaluating one drug or multiple drug combinations; however, only 110 studies had a formal protocol. The clinical studies published in this period were not characterized by having adequate methodological rigor: most studies were case reports and clinical trials with a medium sample size and a heterogenous length of duration (ranging from 3 to 84 months with 47% of the studies not reporting the length). The trial design most commonly used was a self-matching group of patients, and a random method of the assignment was only described in three trials. Three studies used a placebo, three trials used a single-blind or double-blind technique, and 107 of the clinical trials did not report bias or were uncontrolled [67].

Additionally, the description and analysis of results were very loose, heterogeneous, and without adequate rigor. Many of the studies (*n* = 90) did not report whether they used an adjunctive treatment, while a description of demographic characteristics was scarce: only 5% of all the studies had complete demographic characterization. Results such as seizure counts, type of seizure, and side effects were described with observational methods, and results were reported by the percentage of patients improved [67]. This generated important growth in the therapeutic options of the moment, with fewer sedative effects [67,68]. However, the studies’ poor methodological design and the scarcity of effective medications for epilepsy until the 1960s made it easier to demonstrate an improvement in seizure control [68]. During this period, antiseizure medications still used in clinical practice such as phenobarbital, carbamazepine, valproic acid, ethosuximide, and primidone were approved. Some of them were approved by some countries with results obtained from uncontrolled observational clinical studies. However, by the 1970s, controlled studies began to be designed, which expanded approvals by the FDA to include drugs such as carbamazepine or valproic acid [69].

### 4.1. A New Era for the Development of Clinical Trials

In 1962 the Kefauver–Harris Drug Amendment was instated, which meant American manufacturers would have to provide the United States Food and Drug Administration (FDA) with substantial evidence, not only for efficacy but also regarding safety, in order to receive approval for a new drug following “adequate and well controlled investigations, including clinical investigations by experts”. Additionally, this amendment would introduce what we now have come to know as the Phase 1, Phase 2, and Phase 3 structure of clinical trials, marking the beginning of a new era for clinical trials in AEDs [68,70].

In fact, after the 1970s, there was no further development of new AEDs; on the contrary, during this time different clinical studies with a better methodological design were carried out with the existing AEDs. This has provided a better understanding of pharmacokinetics, individualization of doses, therapeutic drug monitoring, drug interactions, clinical response, spectrum of activity, and adverse effects of AEDs. Furthermore, results from many of these studies led to the understanding that polypharmacy is associated with toxicity without improving seizure control, and that monotherapy can achieve seizure control with drug-level monitoring [69].

At the beginning of the 1980s, studies with the existing AEDs continued and the results from these clinical trials allow us to understand the tolerance and efficacy of the first-generation AEDs. For example, a double-blind randomized trial by White et al. [71] comparing phenobarbital, primidone, phenytoin, and carbamazepine sought to identify the antiepileptic medication with the best efficacy and found that phenytoin had the best antiseizure activity. In addition, the authors proposed the maximum dose for each one of the medications in the trial to provide the patient with the best outcome measured in seizure activity [69]. The definition of second-generation AEDs varies depending on the exact year they were introduced; however, molecules such as felbamate, one of the first second-generation AEDs marketed in the United States, was introduced in the year 1993, with many other second-generation AEDs emerging between the end of the 1980s and the early 1990s [72]. Some AEDs such as oxcarbazepine, introduced in the year 1993 [73], were studied in monotherapy compared with carbamazepine as add-on therapy, in placebo-controlled, double-blind, cross-over and fixed-dose trials [68,69]. Second-generation AEDs also had a longer period between the first clinical trial and the first approval. In fact, clinical studies with lamotrigine date back to 1985 and it was approved by regulatory agencies between 1990 and 1995.

### 4.2. Development of Second-Generation AEDs and Future Perspectives

From 1990 until today, clinical trial designs have been characterized by randomized, double-blind, and placebo-controlled trials [69,74]. Clinical trials from 1990 to the present have been novel in implementing: stricter eligibility criteria; parallel group design (replacing the cross design); inclusion of maintenance period; and clinical studies of long-term prognosis [69]. For instance, longitudinal cohorts, where patients are followed for 30 years, allow a wider observation time than the initial studies [75]. However, the use of a placebo has important considerations. First, exposing individuals to placebo therapy may be unethical and increase the risk of sudden death associated with epilepsy; therefore, many second-generation AEDs are developed as adjuvant therapy, making the generation of designs that minimize placebo exposure one of the greatest challenges second-generation AEDs face. Some of the strategies used have been time-to-event designs, in which participants withdrew from the study once they experienced a predetermined number of seizures [69].

Since 1989, the list of antiseizure drugs has increased considerably, with approximately 18 new molecules having entered the market [75]. With these novel molecules, it was hoped that patients with difficult-to-control epilepsy and refractory epilepsy could improve their clinical outcomes; however, only a small proportion of subjects with refractory epilepsy achieved seizure control. Nevertheless, second-generation AEDs continue to have a better safety profile compared to first-generation antiepileptics [69].

In recent years, clinical trials in AEDs have aimed to develop antiseizure drugs for orphan diseases or molecules that act directly on epileptogenic mechanisms [59]. Some of these new molecules include cannabidiol (approved in 2018) for Dravet and Lennox Gastaux syndrome; everolimus (approved in 2017) for focal seizures associated with tuberous sclerosis complex; felbamate (approved in 1993) for seizures associated with Lennox Gastaux syndrome; and rufinamide (approved in 2007) for Lennox Gastaux; and vigabatrin (approved in 1989) for infantile spasms [75]. The design of antiseizure medications, in most cases, has aimed at controlling seizures; however, the design of new molecules for epilepsy should target the etiology of epilepsy [75]. For instance, inflammatory pathways in epilepsy have been the target of a wide range of drugs such as anakinra, adalimumab, or minocycline that act as antagonists of IL-1R1, TNF alpha, and microglia, respectively [76].

## 5. Artificial Intelligence (AI) in AED Development: Challenges and Avenues

Over the last ten years, technology has become an ally for science and has, synchronously with medical knowledge, been able to offer therapeutic strategies for diseases that in the past were intractable. The growing use and applications of artificial intelligence techniques have opened innovative paths in biomedical research, including the discovery of AEDs. In this section, we will address the application of artificial intelligence techniques in the development of AEDs with the aim of articulating the gaps in the current methodology and possible future applications.

### 5.1. AI and Drug Design

Drug development is a time-consuming, expensive task with a high probability of failure that could be optimized with artificial intelligence (AI) techniques [77]. Drug development requires the analysis of complex biological systems, which in our era involves processing heterogeneous sources of data and a great amount of information, including biomedical data from wearable devices, and genomic, metabolomic, and proteomic profiles [78]. Researchers have taken advantage of data challenges in drug development to think about the problem from another perspective, using AI as an advantageous strategy. AI could be defined as an advanced computational method for scientific discovery or scientific understanding [79]. In drug development, AI could be a promising tool as it can analyze great amounts of information, reveal hidden molecule properties, and simulate different conditions with adjusted parameters, leading to discoveries related to multiple processes underlying complex phenomena [80].

**Figure 2:**
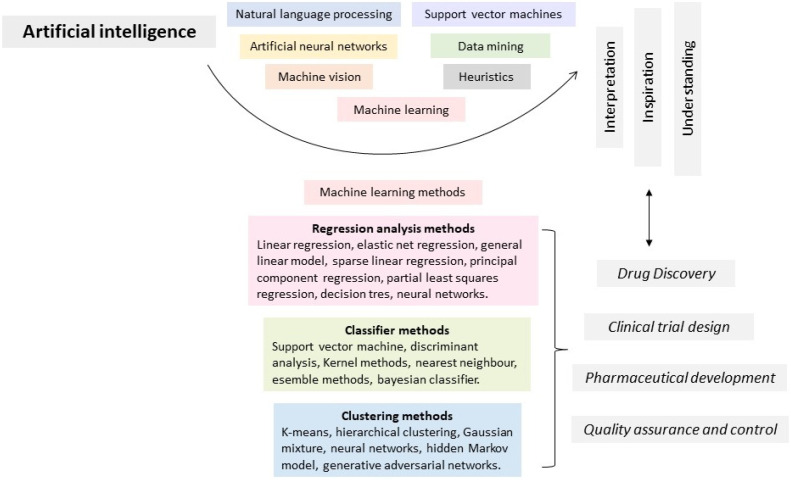
Machine learning and drug discovery.

Machine learning (ML) is one of the most common AI methods used in healthcare [81]. ML uses statistical methods to create a model capable of generalizing concepts using a training dataset, a process that is possible if the model learns information that could later be applied to new data that it has not seen before. The model’s performance is assessed through performance metrics that allow it to select the most efficient model [82], creating a methodological advantage by opening multiple possibilities and probabilities of model selection in drug development. Indeed, ML techniques are useful in all phases of drug development, for drug discovery, clinical trial design, pharmaceutical product development and management, quality assurance, and control [83] (Figure 2). Adopting these techniques in the research phases of drug development could decrease the failure rate that results from associated factors such as lack of efficacy, undesirable toxicity, inconsistent properties, and commercial difficulties [84].

Recently, ML has been predominantly used in drug development for target identification and validation, lead compound discovery, synthesis, protein–ligand interactions, and predictive biomarker discovery [85]. With these techniques, it has become easier to identify target protein structures and design novel drugs from those targets. Usually, the first step is the extraction of ligands and protein features through the information available in online repositories. This procedure allows the modeling of possible active sites for a specific ligand. The screened compounds are exposed to conditions with different physicochemical properties. Finally, leading compounds undergo in vitro and in vivo assays for validation [86]. Another widely used strategy is modeling through quantitative structure–activity relationships (QSAR). This technique identifies relationships between chemical structures and biological activity in the studied compounds using mathematical models [87]. There are multiple ML methods that have been successfully applied at different drug development stages, and these are presented in Table 2.

The use of ML in drug development has limitations because it is a novel area that uses multiple methods and analysis strategies. The main applications have been found in the development of antimicrobial and antineoplastic drugs [108]. In nervous system diseases, drug development is limited due to a higher attrition rate compared to other drugs [109]. The next section will discuss novel drug development strategies, mainly focused on ML techniques and AED development.

### 5.2. Novel Approaches in AED Technology

The development of new drugs for nervous system diseases has great challenges, which could explain the high failure rate in drug discovery [77]. Epilepsy is not the exception, as it comprises heterogeneous conditions that challenge its identification and treatment. AED development has critical needs related to the heterogeneity of epilepsy conditions, gaps in translational research, and the considerable percentage of patients who are refractory to treatment [110]. In response, precision medicine intends to integrate biodata to predict an individually variable response to epilepsy interventions [111]. With precision medicine, it is possible to obtain a holistic view of epilepsy as well as integrate multiple factors in order to understand the phenomenon from the genotypic and molecular substrates that condition a seizure phenotype and propose innovative therapeutic targets.

**Figure 3:**
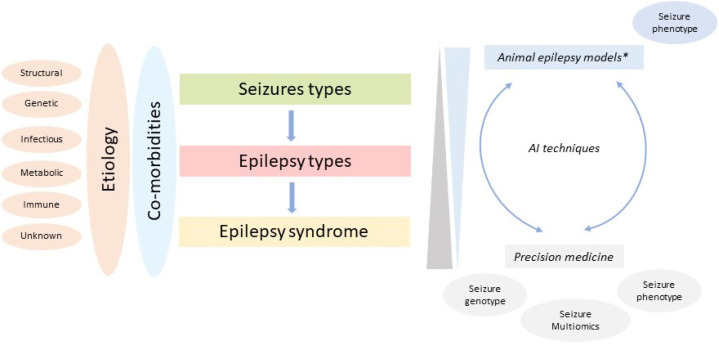
Avenues in AED development: AI techniques as a bridge between animal epilepsy models and precision medicine. * MES, scPTZ, kindling models. Adapted from Scheffer et al. [116].

Pharmacology repurposing (PR) is a growing strategy that was potentiated during the SARS-Cov2 outbreak [112]. PR seeks to identify new uses of previously studied molecules with well-known pharmacological, pharmacokinetic, and toxicological properties. In the last five years, it has been a key method applied in AED development. Applying ML analysis in PR is advantageous because computational methods could detect relationships between various types of biodata, thereby reducing time and costs [113]. Integrating AI methods and PR, some studies have identified molecules widely used in clinical practice that could have potential as AEDs such as doxycycline, metformin, nifedipine, and pyrantel tartrate [114,115]. In fact, AI techniques and PR could be a bridge between approved experimental methods based on seizure phenotypes in animal models and novel precision medicine strategies (Figure 2 and Figure 3).

In the clinical phases of AED drug development, novel approaches, and AI methods could help in the first stage of discovery and development, target validation, compound screening, and optimization of the lead compound. As seen in Table 1, multiple methods and tools could be applied in this stage. As an advantage, the data sources come from online, open repositories with a large amount of information. The challenge is to develop target identification and prioritization based on gene-disease associations, and design compounds with desirable properties with ligand-based screening. In preclinical research, biomarkers are essential for the identification, classification, and prediction of clinical endpoints. AI techniques at this phase could integrate information and aid in decision making [117,118]. In phase I, AI could aid in dose escalation and evaluating toxicity using methods for relating the structure, activity, and mode of action. In phases II and III, it can confirm dose efficacy and toxicity. Finally, for drug review and post-marketing surveillance and safety monitoring, ML models could predict and follow up on specific concerns related to treatment response and adverse events [119].

## 6. Limitations and Future Directions

Advancing drug development in AEDs necessitates a shift from serendipitous discoveries to data-driven research, incorporating an innovative perspective with an integrative research approach focused on understanding the underlying mechanisms. AI techniques have the potential to be the cornerstone tool for integrating translational and precision medicine, leading to the development of a new generation of AEDs with unprecedented efficacy and safety profiles. The use of AI techniques in drug development has highlighted challenges in reproducibility and external validation, to overcome these difficulties, a trans-sectoral collaborative approach is desirable, aiming to establish universal standards that address these challenges effectively.

## 7. Conclusions

Effective antiepileptic treatment has been used for over a century; yet, a substantial number of patients in LMICs are not receiving treatment, thus increasing the morbidity and mortality associated with this disease. Additionally, when receiving treatment, practical guidelines and recommendations for patients in LMICs usually include AEDs that are nowadays not recommended in HICs, such as phenobarbital, an AED that has been proven efficient in controlling seizures but whose adverse effects are still a matter of concern. The undertreatment of epilepsy in LMICs, either due to low accessibility or high-cost therapies, is of major concern and should be targeted in order to reduce mortality and the burden of disease. Development of new AEDs should consider and thus overcome these barriers to guarantee efficient and accessible treatment.

The development of new AEDs presents significant challenges in terms of effectiveness and safety. Approximately one-third of epileptic patients do not respond to treatment, which raises questions about the underlying mechanisms of the disease. Epilepsy, being a multifactorial and complex inherited disease, requires the identification and prioritization of targets based on interactions between “multiomics” and health determinants. Therefore, in preclinical research, experimental designs based on biomarkers serve as the initial step in designing compounds with desirable properties and conducting ligand-based screening [120,121].

In relation to the clinical development of AEDs, it can be concluded that the earliest discoveries were made through serendipity or through the conduct of case series or observational studies, which inherently possess methodological limitations. Over time, randomized controlled trials (RCTs) gained prominence for the development of “new” AEDs, which we consider should continue to be the gold standard for the development of future medications for managing this condition. These should be supplemented with phase IV pragmatic clinical studies that confirm the effectiveness and safety of these medications in real-world conditions.

Translational medicine has the potential to revolutionize the development of AEDs by proposing methodologies that consider disease biomarkers, pharmacodynamic responses, and compound–target interactions within the same trial [120,121]. Additionally, precision medicine, which places the patient at the center of care, aims to go beyond symptom management and focuses on individual etiopathological mechanisms [122,123]. Taking into account the epilepsy diagnostic workup is crucial for the development of new AEDs [122,123]. Recent trends in utilizing AI techniques in healthcare have demonstrated their potential in discovering new possibilities for epilepsy management. Methods such as automating processes, analyzing large datasets to identify novel drug candidates, and discovering biomarkers to optimize drug development phases can bridge the gap between translational and precision medicine in the development of AEDs [124].

## Figures and Tables

**Table 1:** Comparison between clinically validated animal seizure and epilepsy models.

Animal Model	Species	Seizure Phenotype	Human Correlate	Clinical Validation	Predictive of AED Toxicity	AEDs Showing Efficacy	Mechanism of Action Identified
Maximal electroshock stimulation (MES)	Mice/rats	Tonic extension seizure	Generalized tonic–clonic seizures.	Identification and development of phenytoin.	No	– Carbamazepine – Phenytoin – Oxcarbazepine – Valproic acid – Phenobarbital – Felbamate – Gabapentin – Lamotrigine – Lacosamide – Topiramate – Zonisamide	– Na+ channel blockers – Enhanced slow inactivation of voltage-gated Na+ channels – K^+^ channel activators – NMDA receptor antagonists – AMPA receptor antagonists – α2δ ligands
Subcutaneous pentylenetetrazol (scPTZ)	Mice/rats	Minimal clonic seizure	Generalized myoclonic seizures and spike–wave seizures.	Discovery of trimethadione, phensuximide, and ethosuximide.	No	– Ethosuximide – Valproic acid – Phenobarbital * – Benzodiazepines – Felbamate – Gabapentin – Tiagabine * – Vigabatrin *	– T-type Ca^2+^ channel blockers – Allosteric GABA_A_ receptor modulators – GABA transport blockers – GABA transaminase inhibitors – α2δ ligands – mGluR modulators
Kindling models	Rats	Limbic seizures	Partial seizures.	Only model to correctly identify antiseizure activity of levetiracetam.	Yes	– Carbamazepine – Phenytoin – Phenobarbital – Valproic acid – Benzodiazepines – Felbamate – Gabapentin – Lamotrigine – Topiramate – Zonisamide – Levetiracetam – Vigabatrin – Lacosamide	– Na^+^ channel blockers – Enhanced slow inactivation of voltage-gated Na^+^ channels – K^+^ channel activators – AMPA receptor antagonists – GABA receptor modulators (for example, barbiturates and benzodiazepines) – α2δ ligands – SV2A ligands

* These AEDs block clonic seizures induced by scPTZ but are inactive against generalized absence seizures and may exacerbate spike–wave seizures. AED: antiepileptic drug; AMPA: α-amino-3-hydroxy-5-methyl-4-isoxazole propionic acid; GABA: γ-aminobutyric acid; GAERS: genetic absence epileptic rat of Strasbourg; mGluR: metabotropic glutamate receptor; SV2A: synaptic vesicle glycoprotein 2A. Adapted from Bialer M, White HS. Key factors in the discovery and development of new antiepileptic drugs [1].

**Table 2:** Machine learning methods used in drug discovery. Adapted from Gupta et al. [87].

Tool and Software	Method	Features	Performance Metric	Objective
LS-align: Algorithm evaluating ligand structural alignment [87].	Machine learning	Generates atom-level structural alignments of ligand molecules.	AUC	Structure–ligand identification
		Input: query structure, template structure, initial alignment. Output: final alignment, final alignment score.		
LigGrep: Tool identifying docked poses in specified receptor/ligand interactions [88].	Machine learning	Prioritizes candidate small molecule ligands using computer docking.	AUROC and pAUROC *	
		Input: docked-compound files for drug target receptor and candidate ligands. Output: names of candidate compounds with poses that satisfy all user-defined filters.		
AutoGrow4 Generates novel drug-like molecules and optimizes pre-existing ligands [88].	Genetic algorithm	Using a genetic algorithm draws on a population of seed molecules to create a new population of potential ligands. Ranks the candidates by calculated fitness.	Docking score (NNScore1, NNScore2), ligand efficiency, diversity score.	
		Input: first generation of independent seed pools formed from high-scoring compounds and diverse compounds. Output: last generation of selected seeds ranked by fitness scores.		
DLIGAND2 [89]	Distance scaled	Predicts protein–ligand binding affinity based on a distance-scaled, finite, ideal gas reference (DFIRE) state.	AUC, EF	
		Input: residue-specific types for protein atoms and a large protein structural dataset for training. Output: binding affinity prediction using either native or docking-predicted complex structures.		
StackCBPred [90]	Machine learning	Predicts structural properties of amino acids to effectively train a stacking-based machine learning method for the accurate prediction of protein–carbohydrate binding sites.	AUC ROC ACC F1 score	
		Input: protein sequence. Output: predictors of protein–carbohydrate binding sites.		
LSA [91]	Machine learning	Computes the similarity of two molecular structures by considering the contributions of both overall similarity and local substructure match.	AUC	
		Input: three-dimensional molecular structures with substructure focus; computing the similarity score based on superimposing. Output: similarity of two molecular structures by considering the contributions of both overall similarity and local substructure match.		
ProPose [92]	Incremental construction algorithm	The combination of ligand- and receptor-based methods steers the virtual screening by ranking molecules according to the similarity of their interaction pattern with known ligands.	N/A	
		Result: energy torsion angle for incremental molecule construction within an active site of a selected receptor.		
TrixX [93]	Machine learning	Structure-based molecule indexing for large-scale virtual screening in sublinear time is among the fastest virtual screening tools currently available.	Enrichment behavior	
		Input: compounds, parameters, receptors. Output: compound placement score.		
DEEPScreen [94]	Convolutional neural networks	High-performance drug target interaction prediction. Used in the fields of drug discovery and repurposing for in silico screening of chemogenomic space.	F1 score, MCC	
		Input: 2D images of compounds Output: binary classification		
QSAR modeling				Structure and biological activity relation
ChemDes [95]	Pybel, CDK, RDKit, BlueDesc, Chemopy, PaDEL, and jCompoundMapper	An integrated web-based platform for the calculation of molecular descriptors and fingerprint computation.	AUC	
		Input: molecules. Output: QSAR, virtual screening, ranking, ADME/T prediction.		
ChemGrapher [96]	Deep learning	Optical graph recognition of chemical compounds. Produces all information necessary to relate each component of the resulting graph to the source image.	AUC	
		Input: image. Output: molecular graph structure.		
ANFIS [97]	Neuro-fuzzy modeling and principal component analysis	Evaluates physicochemical descriptors of certain chemical compounds for their appropriate biological activities in terms of QSAR models with the aid of an artificial neural network (ANN) approach combined with the principle of fuzzy logic.	AUC	
		Input: fuzzy linear regression. Output: adaptive neuro-fuzzy inference (ANFIS).		
DrugNet [98]	Machine learning	Simultaneous integration of information about diseases, drugs, and targets can lead to a significant improvement in drug repositioning.	AUC	
		Input: drugs. Output: repositioning of drugs with ranked lists for a given disease.		
RepCOOL [99]	Random forest classifier	The potency of the proposed method is in detecting true drug–disease relationships.	AUC and ROC	
		Input: extracting primary data. Output: suggested new drug.		
GIPAE [100]	Gaussian interaction profile kernel and autoencoder	Computational drug repositioning is designed to identify new indications for existing drugs. The batch normalization layer and the full-connected layer are introduced to reduce training complexity.	AUC and ROC, F1 score	
		Input: drug and disease Gaussian interaction. Output: drug and disease association prediction.		
DrPOCS [101]	Machine learning	Predicts potential associations between drugs and diseases with matrix completion.	AUC, F1 score	
		Input: drug, disease. Output: association prediction.		
RCDR [102]	Collaborative filtering model	Prioritizes candidate drugs for diseases.	AUC and ROC	
		Input: drug set, disease set, drug–disease association. Output: predicted association matrix.		
Chembench [103]	Quantitative structure–activity relationship (QSAR) modeling methods	Tools and services for computer-assisted drug design and computational toxicology.	Correct classification rate, accuracy, negative predictive value, positive predictive value	Physicochemical properties
		Input: standardized chemical compounds. Output: dataset visualization, modeling, model validation, virtual screening.		
mCSM-lig [104]	Machine learning models, Platinum database	Effective in predicting a range of chemotherapeutic, antiviral, and antibiotic resistance mutations, providing useful insights for genotypic screening and guiding drug development.	AUC and ROC, precision, accuracy	
		Input: mutation in protein–ligand complexes. Output: mCSM—lig signature.		
DendPoint [105]	Machine learning and principal component analysis	Used to guide dendrimer construct design and refinement before embarking on more time-consuming and expensive in vivo testing.	AUC and ROC	
		Input: pharmacokinetic parameters. Output: predictive values (half-life, clearance, %Dose urine, %Dose liver).		
ProTOX-II [106]	Molecular similarity, fragment propensities, and machine learning	Webserver for the prediction of toxicity of chemicals. Predicts acute toxicity, hepatotoxicity, cytotoxicity, carcinogenicity, mutagenicity, and immunotoxicity.	AUC, balanced accuracy, Kappa index	Mode of action and toxicity
		Input: SMILES string, drawing of the chemical structure, compound name (pubchem). Output: median lethal dose, toxicity class, average similarity with three most similar toxic compounds.		
ADMETlab [107]	Designed based on the Django framework in Python	Early drug-likeness evaluation, rapid ADMET virtual screening or filtering, and prioritization of chemical structures.	ACC, SP, SE, AUC, and ROC	
		Input: SMILES string, drawing of the chemical structure. Output: ADMET profile.		

* AUC: area under the curve; AUROC: area under the receiver operating characteristic; ACC: selected accuracy; EF: enrichment factor; MCC: Matthew’s correlation coefficient; ROC: receiver operating characteristic; SE: sensitivity; SP: specificity.

## Data Availability

Not applicable.
